# Child neglect in one-child families from Suzhou City of Mainland China

**DOI:** 10.1186/1472-698X-14-8

**Published:** 2014-03-25

**Authors:** Jing Hua, Zhe Mu, Bright I Nwaru, Guixiong Gu, Wei Meng, Zhuochun Wu

**Affiliations:** 1 Shanghai First Maternity and Infant Hospital, Tongji University School of Medicine Shanghai, Shanghai, China; 2 Shanghai Urban Environmental Meteorology Center, Shanghai, China; 3 The Medical School of Tongji University, Shanghai, China; 4 School of Health Sciences, University of Tampere, Tampere, Finland; 5 Pediatrics Research Institution of Suzhou University, Suzhou, China; 6 Department of Epidemiology, Public Health School, Fudan University, Shanghai, China; 7 Key Laboratory of Public Health Safety, Ministry of education, Shanghai, China

**Keywords:** Child neglect, One-child policy, China

## Abstract

**Background:**

The one-child policy introduced in China in 1979 has led to far-reaching changes in socio-demographic characteristics. Under this policy regime, each household has few children. This study aims to describe the prevalence of child neglect in one-child families in China and to examine the correlates of child neglect.

**Methods:**

A cross-sectional study of 2044 children aged 6 to 9 years and recruited from four primary schools in Suzhou City, China was conducted. Neglect subtypes were determined using a validated indigenous measurement scale reported by parents. Child, parental and family characteristics were obtained by questionnaires and review of social security records. Linear regression analyses were performed to estimate the associations between these factors and the subtypes of child neglect.

**Results:**

The prevalence of child any neglect was 32.0% in one child families in Suzhou City, China. Supervisory (20.3%) neglect was the most prevalent type of child neglect, followed by emotional (15.2%), physical (11.1%), and educational (6.0%) neglect After simultaneous adjustment to child and family characteristics and the school factor, boys, children with physical health issues and cognitive impairment, younger and unemployed mother, were positively associated with neglect subtypes. We also found that parents with higher education and three-generation families were negatively associated with neglect.

**Conclusion:**

The rates of child neglect subtypes vary across different regions in China probably due to the different policy implementation and socio-economic levels, with a lower level of physical and educational neglect and a higher level of emotional neglect in this study. The three-generation family structure was correlates of neglect which may be unique in one child families. This indicates that future intervention programs in one-child families should target these factors.

## Background

Child neglect is the failure of a parent or a caregiver in a parenting role to meet the basic needs of the child, which poses a major threat to the child’s healthy growth and well-being [1]. The National Incidence Study of Child Abuse and Neglect [2,3] in the United States defined neglect as encompassing physical neglect, child abandonment and expulsion, inadequate supervisory neglect, emotional neglect, and educational neglect by parents, parent substitutes, or other adult caregivers of children. The prevalence of child neglect varies across countries. The rates of neglectful behavior of parents recalled by university students in 17 nations ranged from 3.2% to 36% [4]. The prevalence of supervisory and physical neglect reported by a national sample of adolescents in the United States were 42% and 12%, respectively [5]. In the United Kingdom, 6% and 5% of young adults reported the absence of care and supervision, respectively [6].

There is evidence that children’s and familial characteristics are the main determinants of child neglect, including maternal education [7], maternal age [8], family structure [9], and employment instability and poverty [10]. Other studies have also shown that prematurity, prolonged hospitalization during childhood, mental impairments and developmental disabilities [11] of children were positively associated with child neglect.

Despite its negative effects and accordingly, warranting active research, studies on child neglect in China are scarce. One reason for the limited studies may be the lack of appropriate validated instruments because cultural norms concerning neglectful behaviors could vary from society to society. Recently, Pan et al have developed and validated the Scale on Child Neglect in Urban China (SCNUC) [12-14]. The instrument’s cultural appropriateness was examined through focused (semi-structured) interview with China-born mothers. The results showed that SCNUC is culturally sensitive, although there would be no assurance that the instruments would be useful for cross-cultural comparisons [12,14]. Researches using SCNUC have reported the rates of neglect vary across different regions from 11.6% in Guangzhou City (among children aged 3-6 years old in South China) to 50.0% in Yinchuan City (among children aged 6-8 years old in western China) in Chinese language literature [15-20]. The correlates of childhood neglect such as low parental education, young maternal age, and children with physical and cognitive problems were also reported in these studies [15-20].

Additionally, the Chinese State Council launched the one child policy in 1979 [21], which is almost unique globally. Under this policy regime, each household has few children. As a result, each child, often described as the ‘little emperor’ or ‘little sun’ [22], has increasingly become the center of attention in the household and has become more precious to parents than ever. Recently, Zhang et al [19] have found that the one-child and multiple-child families differ in their level of neglect in preschool children based on a relatively small sample in Guangzhou city of China. The one-child families were characterized by lower prevalence of neglect, although the levels of specific neglect subtypes were not reported in that study. Because only 17% of Chinese elders currently have some form of pension and more than 70% rely only on the support of their children [23], the child in one-child families was regarded as the “only hope” of giving their parents support when they become old [23]. Parents from one-child families attach high value to their children, hence may take steps to protect them from physical harm and foster in them socially desirable and culturally approved values. In addition, by the end of 1992, employed females have made up more than 70 percent of all women over 15 years old in China [24]. Mothers spent limited time to take care of their children although most of them were still the primary caregivers under the influence of the traditional Chinese culture. Under the socio-cultural regime, mothers in one-child families may pay great attention to children’s education and physical needs possibly at the cost of providing them with adequate emotional environment.

To understand the patterns of child neglect in one-child families in China, we therefore conducted a pilot study in Suzhou City in Yangtze River Delta, one of the most developed areas in China with rigorous operation of the ‘one-child’ policy. We hypothesized that the prevalence of child neglect in one-child families may be lower than that in multi-child families. There are cross-region difference in child neglect due to the different policy implementation and socio-economic levels in China. We also hypothesized that there would be a lower level of physical and educational neglect and a higher level of emotional neglect in our sample. Moreover, we expected that the correlates of neglect found in this sample would be similar to those reported in other studies. However, unique factors in one-child families associated with child neglect may also exist. For example, three generation families under one-child policy are structured as 4-2-1(four grandparents, two parents and one child), and this structured may decrease the likelihood of child neglect with more adult family members taking care of their “only one” child [25]. We aimed: (1) to describe the prevalence of child neglect and its subtypes among children from one-child families in Suzhou City, China, and (2) to examine the correlates of child neglect in one-child families in this context. Because the measurement for child neglect is different in each age group and its determinants may vary across age groups [13], the focus of the current study is on child neglect in children aged 6-9 years old. Child neglect in other age groups will be reported in another parallel study.

## Methods

### Study design and participants

Of the three districts (Pingjiang, Canglang, Jinchang) in Suzhou Ancient City (urban area of Suzhou), Canglang district was randomly sampled for the study. Of the 19 public primary schools in this district, five were randomly selected in the study using stratified random sampling. The size of the sample in each stratum is taken in proportion to the size of the stratums (of these schools, 40% were key primary schools, and 60% were general primary schools). Only one school refused to participate in the study because the study period coincided with the school sports meetings. Therefore, a total of 2230 children’s caregivers in four primary schools were recruited, which involved filling out a self-administered questionnaire. The survey was conducted from October to November 2006. Teachers of the participating schools handed out the questionnaires to the children’s primary caregivers (biological mothers 89%, biological fathers 6%, and grandparents 5%) at the parent-teacher meeting. The primary caregivers were asked to fill out the questionnaire individually in half an hour at end of the conference according to the attached illustration, then place it in a sealed envelope and return to the children’s teachers. Only the researchers involved in the study could open the envelope when it was returned. The questionnaire comprised of 91 question items which asked information related to child neglect. Of the 2230 questionnaires distributed, 2166 (97.1%) were returned. Of the returned 2166 questionnaires, 117 (5.6%) were excluded from the analysis due to missing values, and five subjects were excluded because their families had more than one child. The final sample used for the analysis comprised 2044 children. All of these children belong to Han ethnic group (the majority of the population in the Yangtze Triangle Delta) and come from one child families. The study was approved by the local Education Board and Ethics Committee of Children’s Hospital of Suzhou University. Participation in the study was voluntary. Oral parental/guardian consent and students’ assent were obtained before the commencement of the study. All the information acquired was kept confidential and was only accessible to the researchers.

### Measures

In this study, the child neglect was measured by SCNUC [12-14]. The internal consistency (Cronbach’s alpha and split-half coefficients were 0.94 and 0.88 respectively), test-retest reliability (Pearson correlation coefficient were 0.93), and construct validity of SCNUC (Item-total correlation were above 0.93) were good. Four subtests of SCNUC were used in this study, which contained physical neglect (12 items), emotional neglect (11 items), educational neglect (16 items), and supervisory neglect (11 items). Physical neglect refers to caregivers not taking care of child’s basic needs (such as “Neglect to provide breakfast for child”, and “Neglect to provide the individual-used toiletries for children” and the reversed item of “Buy fruit and vegetables for child every day”). Educational neglect refers for the caregiver’s failure to provide educational opportunity to the child (such as “Neglect the child’s schooling”, and the reversed item of “Pay attention to the child’s performance during school time”). Emotional neglect refers to the caregiver’s failure to provide the child with an emotional environment that allows adequate psychological, cognitive and physical development (such as “Break child’s toy or other things when feel angry”, “Scold and hit the child before others”, and the reversed “Embrace or kiss the child”). Supervisory neglect describes caregiver’s failure to protect the child from physical harm or danger (such as the reversed items of “Teach the child how to protect against fire, power line and gas” and “Teach child how to cross the road”). Caregivers were asked to indicate how often they had conducted the listed neglectful behavior with a 4-point Likert scale (1 = never, 2 = occasional, 3 = usual, 4 = constant) in the past one year. The scores of the subscales represented the sum of their corresponding items (the reversed items were reversely scored). A child was identified as being neglected when the scores of the subscales were above the cut-off scores (25 for physical neglect, 25 for emotional neglect, 35 for educational neglect, 25 for supervisory neglect) which were 90 percentile of the national norms based on the data from 8001 children in 28 cities of China [12-14]. The more sever the neglectful behavior, the higher the score. Additionally, we also tested the reliability and validity of the four SCNUC subscales in this sample. The results showed that the Cronbach’s alpha coefficients of the physical, emotional, educational, supervisory neglect subscales (internal reliability) were 0.87, 0.82, 0.91 and 0.85, respectively. Split-half coefficients of the four subscales were 0.83, 0.85, 0.89 and 0.81, respectively. The item-total correlations of the four subscales (construct validity) were all above 0.85. For assessment of the criteria-related validity, 200 families (10% of the total sample) were randomly selected to take part in semi-structured interviews for identifying neglectful behaviors a week after filling out the SCNUC. Correlation coefficients of four SCNUC subscales between the results of interview and results determined by cut-off scores were 0.81,0.83,0.89 and 0.79, respectively, indicating the criteria-related validity of the subscales were fair. These results showed that the four subscales of SCNUC could be applied in our study.

We obtained the information about children’s and families’ characteristics from the questionnaires, including the child’s and mother’s age; child’s sex; mother’s employment (whether or not the mother was employed in the past two years); maternal health issues (whether or not the mother had a physical or psychological illness in the past two years); parental education (whether or not the parents obtained a degree of higher education); family structure (families with three generations, nuclear family, single mother or father); and domestic per-capita disposable income of every month in the past year. Great importance was attached to maternal features in this study because mothers are typically the primary caregivers of children in Chinese society and primarily responsible for failures to provide adequate care. “Family with three generations” refers to the child living with his/her biological parents and grandparents, and it was usual among Chinese families due to the traditional family culture. The Families with three generations are structured as 4-2-1(four grandparents, two parents and one child) in one-child families. “Nuclear family” indicates that the child was living with his/her biological parents, and “single mother or father” refers to the child living with one of his/her biological parents.

Because almost all school-aged children and their parents took part in the municipal medical insurance in Suzhou City, the information about children and their parents’ history of diseases could be obtained from the medical records provided from the local medical insurance department of social security bureau. In this study, based on children and their parents’ basic information including name, gender, and date of birth (obtained from the questionnaires), their medical records were found in the medical information system of local medical insurance department of social security bureau. The children or parent’s health issues (whether or not they had stayed in the hospital in the past two years) and whether there was any cognitive impairment (a variety of impaired cognitive functions such as Attention Deficit Hyperactivity Disorder, Autism Spectrum and Developmental coordination disease) were measured according to their medical records.

### Statistical analysis

One-way ANOVA and independent sample t-test were used to compare the means of neglect sub-scores. And the Least Significant Difference (LSD) analysis was used for post-hoc tests of the significant ANOVAS. Linear regression analysis was used as the main statistical technique to investigate the associations between the independent variables (child’s and family characteristics) and the dependent variable (scores of subscales for neglect subtypes). All the studied independent variables (child’s and family characteristics) were simultaneously included in the linear regression model. Because the Variance Inflation Factor (VIF) of each independent variable in the model was less than 10, the multicolinearity was not statistically significant. Therefore, the colinearity was not considered in the model. The analysis was carried out using SPSS for Windows Version 17.0. A *p*-value of <0.05 was considered statistically significant.

## Results

### The rates of neglect subtypes

Mean score of physical neglect was 23.351 (range from 15 to 46) with a Standard Deviation (SD) of 4.915. Mean score of emotional neglect, educational neglect, and supervisory neglect were 23.124 (SD = 3.905, rang = 14 to 42), 30.513 (SD =7.636, rang = 18 to 61), and 31.232 (SD = 6.150, rang from 19 to 44) respectively. Table 1 shows the mean scores of subscales for neglect subtypes by children and family characteristic. According to the scores of neglect subtypes, of 2044 children, 20.3% were identified as the supervisory (95% CI 19.2% to 22.5%) neglect, followed by emotional (15.2%, 95% CI 14.1% to 17.4%), physical (11.1%, 95% CI 9.7% to 12.0%), and educational (6.0%, 95% CI 5.0% to 7.0%) neglect in one child families of Suzhou. The prevalence of any neglect (the child had experienced at least one type of neglect) was 32.0% (95% CI 30.1% to 34.0%). Moreover, 8.3% (95% CI 7.1% to 9.5%) had experienced 2 neglect subtypes, while 4.3% (95% CI 3.4% to 5.2%) had experienced ⩾3 subtypes of neglect.

**Table 1:** The scores of subscales for neglect subtypes according to child and family characteristics (n = 2044)

**Characteristic**	**N**	**Physical neglect**	**Emotional neglect**	**Educational neglect**	**Supervisory neglect**
		**Mean (SD)**	**Mean (SD)**	**Mean (SD)**	**Mean (SD)**
School^a^					
1	263	24.008(5.115)	23.388(3.76)**	31.183(7.529)***	31.065(5.973)
2	194	24.18(5.378)	23.392(3.88)	32.263(7.86)	31.773(6.347)
3	547	23.602(4.837)	23.395(3.757)	31.554(7.118)	31.066(6.217)
4	1040	22.829(4.765)	22.785(4.002)†	29.469(7.741)††	30.707(6.114)
Children’s age^a^					
6	417	22.736(4.659)*	23.103(3.878)	30.755(7.799)	30.633(5.862)
7	789	23.238(4.869)	22.976(3.833)	30.624(7.608)	30.932(6.131)
8	736	23.597(5.045)	23.14(4.008)	30.107(7.479)	31.035(6.257)
9	102	24.255(5.12)	23.422(3.834)	31.588(8.229)	31.775(6.636)
Gender^b^					
Girls	977	23.413(4.925)**	23.317(3.953)*	30.443(7.848)	30.728(6.051)
Boys	1067	22.227(4.908)	22.828(3.837)	30.576(7.44)	31.154(6.234)
Physical health issues^b^					
No	1374	23.274(4.862)	22.929(3.787)*	30.432(7.575)	30.818(6.033)^c^
Yes	670	23.400(5.025)	23.399(4.120)	30.678(7.765)	31.221(6.379)
Cognitive impairment^b^					
No	1557	23.141(4.953)**	22.704(3.812)***	30.083(7.643)***	30.719(6.103)**
Yes	487	23.873(4.757)	24.296(3.953)	31.887(7.459)	31.688(6.246)
Mother’s employment^b^					
Empolyed	1869	23.181(4.836)***	23.014(3.889)**	30.311(7.575)***	30.844(6.134)**
Unemployed	175	24.754(5.512)	23.817(4.010)	32.663(7.978)	32.08(6.22)
Mother’s health problems^b^					
No	1107	22.956(4.861)**	23.026(3.863)	29.778(7.354)***	30.668(6.041)
Yes	937	23.62(4.943)	23.132(3.941)	31.135(7.817)	31.189(6.233)
Higher education of mother^b^					
No	1129	23.905(4.972)***	23.524(3.828)***	31.975(7.649)***	31.281(6.278)**
Yes	915	22.588(4.747)	22.539(3.932)	28.708(7.228)	30.542(5.966)
Higher education of father^b^					
No	973	23.94(5.012)***	23.562(3.983)***	31.786(7.735)***	31.292(6.213)*
Yes	1071	22.748(4.757)	22.648(3.782)	29.356(7.361)	30.64(6.078)
Family structure^a^					
Nuclear family	1483	23.469(4.947)***	23.161(3.868)	30.353(7.640)	30.811(6.142)
Single family	16	21.063(5.053)	22.250(3.550)	28.563(7.090)	28.938(5.053)
Family with three generation	545	22.963(4.800)†	22.895(4.010)	31.006(7.628)	31.389(6.180)

^a^One-way ANOVA.

^b^Independent-samples t test.

*p < 0.05, **p < 0.01, ***p < 0.001.

†*p* < 0.05 , ††*p* < 0.01 (post-hoc test and compare the factor with the first one).

### The correlates of neglect subtypes

Table 2 shows the association between the studied factors and neglect subtypes. After simultaneous adjustment for the child’s and family characteristics and the school factor, the results show that boys experienced less physical neglect than girls (B = -0.437; 95% CI -0.704 to -0.171; *p* = 0.001). Children with physical health issues experience more emotional (B = 0.713; 95% CI 0.339 to 1.086; *p* < 0.001) and educational (B = 1.152; 95% CI 0.423 to 1.882; *p* = 0.002) neglect. On the other hand, children with cognitive impairment suffered from more severe physical (B = 0.628; 95% CI 0.130 to 1.125; *p* = 0.013), emotional (B = 1.454; 95% CI 1.062,1.847; *p* < 0.001), educational (B = 1.393; 95% CI 0.628,2.159; *p* = 0.001) and supervisory (B = 0.865; 95% CI 0.234,1.496; *p* = 0.007) neglect. Younger mothers (B = -0.014; 95% CI -0.133,-0.001; *p* = 0.049) and unemployed mothers (B = 0.857; 95% CI 0.079 to 1.636; *p* = 0.031) were likely to have children with more severe educational neglect. Children of mothers with college education experience less physical (B = -0.642; 95% CI -1.197,-0.087; *p* = 0.024), emotional (B = -0.589; 95% CI -1.027,-0.151; *p* = 0.008), and educational neglect (B = -2.456; 95% CI-3.31,-1.601; *p* < 0.001). At the same time, those whose fathers were college graduates experienced less emotional neglect (B = -0.531; 95% CI -0.962,-0.100; *p* = 0.016). Additionally, children in families with three generations suffered from less physical neglect (B = 0.055; 95% CI -1.039 to -0.135; *p* = 0.023) when compared families with the nuclear family.

**Table 2:** Associations between child and family characteristics and neglect subtypes (n = 2044)

**Characteristic**^**a**^	**Physical neglect**	**Emotional neglect**	**Educational neglect**	**Supervisory neglect**
	**Estimates (95% CI)**	**Estimates (95% CI)**	**Estimates (95% CI)**	**Estimates (95% CI)**
Children’s age	0.322(-0.101,0.745)	-0.007(-0.217,0.204)	-0.226(-0.636,0.184)	0.206(-0.132,0.544)
Gender				
Girls	Ref	Ref	Ref	Ref
Boys	-0.437(-0.704, -0.171)**	0.312(-0.021,0.646)	-0.142(-0.793,0.508)	0.288(-0.248,0.824)
Physical health issues				
No	Ref			
Yes	0.339(-0.135,0.813)	0.713(0.339,1.086)***	1.152(0.423,1.882)**	0.555(-0.046,1.157)
Cognitive impairment				
No	Ref	Ref	Ref	Ref
Yes	0.628(1.125,0.130)*	1.454(1.062, 1.847)***	1.393(0.628,2.159)***	0.865(0.234,1.496)**
Mother’s age	0.002(-0.075,0.079)	0.01(-0.051,0.07)	-0.014(-0.133,-0.001,)*	-0.015(-0.113,0.083)
Mother’s employment				
Empolyed	Ref	Ref	Ref	Ref
Unemployed	1.028(-0.169,2.225)	0.411(-0.203,1.025)	0.857(0.079,1.636)*	0.97(-0.018,1.957)
Mother’s health problems				
No	Ref	Ref	Ref	Ref
Yes	0.213(-0.232,0.658)	-0.168(-0.519,0.183)	0.559(-0.126,1.244)	0.336(-0.229,0.901)
Higher education of mother				
No	Ref	Ref	Ref	Ref
Yes	-0.642(-1.197,-0.087)*	-0.589(-1.027,-0.151)**	-2.456(-3.31,-1.601)***	-0.392(-1.096,0.313)
Higher education of father				
No	Ref	Ref	Ref	Ref
Yes	-0.509(-1.055,0.037)	-0.531(-0.962,-0.100)*	-0.688(-1.528,0.152)	-0.272(-0.965,0.420)
Family structure				
Nuclear family	Ref	Ref	Ref	Ref
Single family	-0.046(-4.932,0.002)	-0.387(-3.337,0.436)	-0.029(-6.203,1.159)	-0.031(-0.110,0.087)
Family with three generations	-0.055(-1.093,-0.135)*	-0.040(-0.732,0.023)	-0.022(-0.350,1.123)	0.038(-5.176,0.893)
Family per-capita income of every month	0.001(-0001,0.002)	0.011(-0.005,0.021)	0.012(-0.003,0.025)	0.009(-0.002,0.010)

^a^Adjusted for children’s school and other variables in the table using linear regression model (method for variable entry and removal:“enter”).

*p < 0.05, **p < 0.01, ***p < 0.001.

## Discussion

The present study sheds light on the regional situation of child neglect in China. Noteworthy contributions of the study include the provision of a regional data on the prevalence of neglect subtypes in one-child families of China for first time in English. The rates of child neglect subtypes vary across different regions in China probably due to the different policy implementation and socio-economic levels, with a lower level of physical and educational neglect and a higher level of emotional neglect in this study. In addition to child’s gender, physical health issues, cognitive impairment, maternal age, employment, health problems, and parental education levels were associated with child neglect, which are similar to factors reported in other studies [10,26-31]. We also found that the 4-2-1 three-generation family structure was correlates of neglect which is unique in one child families of China.

Our study showed a lower prevalence of child any neglect (32.2%) than that of Yinchuan City in western China (50.0%) using the same age bands of SCNUC [20]. However, it is difficult to compare the rates of neglect with those from other studies in China, because most of these studies were conducted in different age groups [15-19]. Supervisory neglect (20.3%) was the most prevalent type of child neglect, followed by emotional (15.2%), physical (11.1%), and educational (6.0%) neglect in one child family of Suzhou City using SCNUC. The study in Yinchuan City [20] also showed that the prevalence of educational neglect (18.1%) was much higher than that of Suzhou City. However, emotional neglect (8.8%) in that study was lower than that of the present study. Yinchuan city is located in ethnic minority autonomous regions (the one-child policy has not been implemented in minority population). Yinchuan city is located in ethnic minority autonomous regions (the one-child families policy has not been implemented in the minority population). The results confirm our expectation that the prevalence of neglect in one-child families were lower than that in multi-child families which is similar to the Zhang’s finding in Guangzhou [19]. Child neglect subtypes may vary across different regions due to the different policy implementation and socio-economic levels in China. The results also partly confirm our hypothesis that parents in one-child families would pay greater attention to their child’s education at the cost of providing them with inadequate emotional environment.

Making a cross-country comparison of neglect prevalence is difficult, because different cultures may have standard and definition of parental behaviors and practices [22], which may directly influence the type of child neglect prevalent in a particular society. In the US, educational neglect of children was reported as the most frequent child neglect subtype, followed by physical and emotional neglect [2,3]. In the current study, educational neglect was the least child neglect subtype, while emotional neglect were the second most common child neglect subtype.

Demographic characteristics that are frequently found to be associated with child neglect, such as children’s sex, parental education, family economic status and structure, were also observed to be associated with child neglect in the present study. A study from Israel found that male children were more commonly involved in all types of maltreatment (including the neglect and abuse) [26]. However, the data from 50 states in United States in 1999 showed no difference between boys and girls with regards to child neglect [32]. In the current study however, we observed that that boys were less likely to experience physical neglect than girls. Our finding may indicate that the traditional culture in which boys tend to be more valued than girls seems to be still operating in Chinese one-child families. Additionally, our results show that children with physical problems were more likely to suffer emotional neglect. Another study reveals that 68% of children with body dysmorphic disorder were emotionally neglected [27]. Our finding that the child’s cognitive impairment is associated with all neglect subtypes might imply that parents from one-child families consider the child as their “only hope”, thereby putting greater expectations to make them culturally desirable. A previous study has indicates that children with physical and cognitive disabilities were 3.4 times more likely to be maltreated than their nondisabled peers [28].

Our results also indicate that children of younger mothers experienced more educational neglect. Maternal age was a strong determinant of all types of child’s maltreatment in a previous study [29]. This may be related to the level of experience and maturity of younger mothers, resulting in the failure to provide adequate education for their child. Furthermore, the association between maternal education and physical, emotional and educational neglect corroborates previous findings [29], which suggest that less educated mothers may more likely neglect their children than those with higher education. Mothers with limited educational background may inadequately meet their child’s basic needs in proper manners. Our results also show that maternal unemployment was associated with educational neglect. It has also been reported that neglectful mothers were less likely to report ever having been employed [30]. Fathers with higher education were only associated with emotional neglect in this study. Recently, studies from western countries emphasize the importance of paternal characteristics in the care of children, showing that father’s characteristics are of significance to childhood neglect [10]. However, in Chinese culture, it is the mother who is responsible more for taking care of her child, including providing emotional and educational support, and our results suggest that maternal factors are associated with more neglect subtypes than those of fathers.

Finally, we did not observe any association between family income and any neglect subtype. This is consistent with other studies [10,31], in which poverty was not associated with physical neglect. Children in 4-2-1 three-generation family experience less physical neglect than those in families with three generations. The 4-2-1 three-generation families with only one child is common (26.7% of this sample) in China according to Chinese culture and under the one-child policy. Compared with the nuclear family, there are much more adults (two parents and four grandparents) who may take care of their “only child”, making child experience less physical neglect.

## Conclusion

In conclusion, child supervisory and emotional neglect were the most common, while educational neglect was the least common among these neglect subtypes in one-child families in China. The rates of child neglect vary across the different regions of China, with a lower level of physical and educational neglect and a higher level of emotional neglect in this study. This deserves greater attention. In support of previous reports, a child’s gender, physical health status, maternal age, employment, parental education level are important correlates of different subtypes of child neglect in China. The family structure which is unique in one-child family was also associated with neglect. As one of the studies in this Chinese context, this study provides a good platform for future intervention programs in one-child families in preventing child neglect, by taking into account the observed family socio-demographic characteristics as potential factors of child neglect. Further studies are also required to investigate whether intervention programs that target improving these elements would improve the childhood neglect and its subtypes.

However, in the present study, the majority of children (above 99.9%) were the “only child” in their family under the rigorous birth control policy in urban area of Mainland China. Therefore, it is impossible to make the comparison between the one-child families and families with more than one child. It is also difficult to compare the neglect prevalence between different nations because there would be no assurance that the instruments we used in this sample would be useful for cross-cultural comparisons. Moreover, the unwillingness to disclose neglectful behavior on the part of the parents may cause the social desirability response bias and underreport children’s experiences of neglect.

## Competing interests

The authors declare they have no competing financial interests.

## Authors’ contributions

JH contributed to design, execution, analysis and writing of the study. GG and WM were responsible for the study design and approval of the submitted and final version. ZW and BIN contributed to revising the paper and approval of the final version. All authors read and approved the final manuscript.

## Pre-publication history

The pre-publication history for this paper can be accessed here:

http://www.biomedcentral.com/1472-698X/14/8/prepub
